# Supplementary material for: Prevalence and correlates associated with early childbearing among teenage girls in Ethiopia: A multilevel analysis
Source: PLoS One. 2023 Aug 8;18(8):e0289102. doi: 10.1371/journal.pone.0289102 (PMC10409268; doi:10.1371/journal.pone.0289102)
Supplement: Attachment — Submitted filename: Rebuttal Letter.docx [file pone.0289102.s001.docx]

We deeply appreciate and thank the reviewers for their insightful comments and recommendations as well as for taking the time to read and suggest our paper.

PONE-D-22-18542

Risk factors and prevalence associated with early childbearing among teenage girls in Ethiopia: A Multilevel Analysis

**Title Modification**: We modified the word “risk factor” by “Correlates” in the title.

**Journal requirements:**

When submitting your revision, we need you to address these additional requirements.

1. Please ensure that your manuscript meets PLOS ONE's style requirements, including those for file naming. The PLOS ONE style templates can be found at

https://journals.plos.org/plosone/s/file?id=wjVg/PLOSOne_formatting_sample_main_body.pdf and

<https://journals.plos.org/plosone/s/file?id=ba62/PLOSOne_formatting_sample_title_authors_affiliations.pdf>.

**Author’s response**: As suggested by the editor, we made an attempt to keep to the guidelines of the PLOS ONE style.

2. We suggest you thoroughly copyedit your manuscript for language usage, spelling, and grammar. If you do not know anyone who can help you do this, you may wish to consider employing a professional scientific editing service.

**Author’s response**: According to the editorial suggestions, we rewrote the entire document using proper grammar.

Whilst you may use any professional scientific editing service of your choice, PLOS has partnered with both American Journal Experts (AJE) and Editage to provide discounted services to PLOS authors. Both organizations have experience helping authors meet PLOS guidelines and can provide language editing, translation, manuscript formatting, and figure formatting to ensure your manuscript meets our submission guidelines. To take advantage of our partnership with AJE, visit the AJE website (http://learn.aje.com/plos/) for a 15% discount off AJE services. To take advantage of our partnership with Editage, visit the Editage website (www.editage.com) and enter referral code PLOSEDIT for a 15% discount off Editage services.  If the PLOS editorial team finds any language issues in text that either AJE or Editage has edited, the service provider will re-edit the text for free.

Upon resubmission, please provide the following:

The name of the colleague or the details of the professional service that edited your manuscript

**Author’s response:** The manuscript has been edited by Abebe Debu Liga, and Akalu Banbeta Tereda.

A copy of your manuscript showing your changes by either highlighting them or using track changes (uploaded as a *supporting information* file)

A clean copy of the edited manuscript (uploaded as the new *manuscript* file).

3. Thank you for stating the following financial disclosure:

“No funding was obtained for this study.”

At this time, please address the following queries:

a) Please clarify the sources of funding (financial or material support) for your study. List the grants or organizations that supported your study, including funding received from your institution.

b) State what role the funders took in the study. If the funders had no role in your study, please state: “The funders had no role in study design, data collection and analysis, decision to publish, or preparation of the manuscript.”

c) If any authors received a salary from any of your funders, please state which authors and which funders.

d) If you did not receive any funding for this study, please state: “The authors received no specific funding for this work.”

### Author’s response: Amended it per the editorial comment (Please see the “Financial Disclosure Statement” section)

Please include your amended statements within your cover letter; we will change the online submission form on your behalf.

4. We note that you have stated that you will provide repository information for your data at acceptance. Should your manuscript be accepted for publication, we will hold it until you provide the relevant accession numbers or DOIs necessary to access your data. If you wish to make changes to your Data Availability statement, please describe these changes in your cover letter and we will update your Data Availability statement to reflect the information you provide.

**Author’s response**: The data used for this study has been in the hands of the first and corresponding authors. So, it will be sent with a reasonable request.

5. Your ethics statement should only appear in the Methods section of your manuscript. If your ethics statement is written in any section besides the Methods, please delete it from any other section.

**Author’s response**: Amended it per the editorial comment.

[Note: HTML markup is below. Please do not edit.]

Reviewers' comments:

Reviewer's Responses to Questions

**Comments to the Author**

1. Is the manuscript technically sound, and do the data support the conclusions?
The manuscript must describe a technically sound piece of scientific research with data that supports the conclusions. Experiments must have been conducted rigorously, with appropriate controls, replication, and sample sizes. The conclusions must be drawn appropriately based on the data presented.

Reviewer #1: Yes

Reviewer #2: Partly

2. Has the statistical analysis been performed appropriately and rigorously?

Reviewer #1: Yes

Reviewer #2: I Don't Know

3. Have the authors made all data underlying the findings in their manuscript fully available?
The [PLOS Data policy](http://www.plosone.org/static/policies.action#sharing) requires authors to make all data underlying the findings described in their manuscript fully available without restriction, with rare exception (please refer to the Data Availability Statement in the manuscript PDF file). The data should be provided as part of the manuscript or its supporting information, or deposited to a public repository. For example, in addition to summary statistics, the data points behind means, medians and variance measures should be available. If there are restrictions on publicly sharing data—e.g. participant privacy or use of data from a third party—those must be specified.

Reviewer #1: No

Reviewer #2: No

4. Is the manuscript presented in an intelligible fashion and written in standard English?
PLOS ONE does not copyedit accepted manuscripts, so the language in submitted articles must be clear, correct, and unambiguous. Any typographical or grammatical errors should be corrected at revision, so please note any specific errors here.

Reviewer #1: Yes

Reviewer #2: No

**Author’s response**: We amended the grammar throughout the paper per the reviewer comment.

5. Review Comments to the Author

Please use the space provided to explain your answers to the questions above. You may also include additional comments for the author, including concerns about dual publication, research ethics, or publication ethics. (Please upload your review as an attachment if it exceeds 20,000 characters)

Reviewer #1: good job. Adolescent reproductive health is always at the center stage because of worsened maternal and newborn health indices. however, you need to describe the ethical processes in data collection or approval to use these secondary data.

**Author’s response**: At the end of the method section, we added the Ethical Approval Statement to the document.

Reviewer #2: Authors must consider engaging an English language expert to help undertake a complete language editing of the entire research work; subject verb agreement, grammatical errors, unusually long sentences and to mention a few.

**Author’s response**: According to the reviewers and editor suggestions, we rewrote the entire document using proper grammar.

The aim of the study is unclear.

**Author’s response**: We attempted to clarify the purpose of this study (please see the third sentence of the abstract's background and the last paragraph in the introduction).
Methodology
Authors should rephrase this statement for clarity--"This study was conducted in 2016 by the Ethiopian Demographic and Health Survey". If secondary data was obtained for this work, it should be clearly stated with appropriate referencing of primary data.

**Author’s response**: We added the relevant reference for it (please see Data source section in the Methods)
Sections of the methodology indicate male and females ages 15-59 years were interviewed. Were males included in this study? Authors should please clarify.

**Author’s response**: Actually, only girls under the age of 19 were considered in this study. This study, on the other hand, was based on survey data collected by the Ethiopian Central Statistical Agency. So the data includes males, females, children, and so on. Therefore, we reported the number of males for this study for this reason. Finally, in order to provide a better understanding for the readers, we removed any information regarding Male and shaped it (please see Data source section in the Methods).

Authors indicated that all girls aged between 15 to 19 years were included in the study and those out of the range were excluded. How was consent obtained for females less than 18 years of age? Was accent sought from this age group in the primary data.

**Author’s response**: We used Ethiopian Demographic and Health Survey data, which was collected by the Ethiopian Central Statistical Agency, as we tried to explain in the preceding comment. As a result, since the data is secondary, there is no need to get consent from the participants concerning the data.

Results
There seem to be copious repetition of the results in the tables in the write up. Authors can consider stating just significant ones and leave the rest in the illustrated tables for readers to peruse.

**Author’s response**: We amended it throughout the paper per the reviewer comment.
Discussion
In-text referencing of CSA, 2016 should be properly done.

**Author’s response**: Amended it per the reviewer comment.

Can authors identify some study limitations and how their study design or approach sought to curtail the limitations.

**Author’s response**: Yes, when using primary data as the research analysis, researchers can find solutions to the limitations. However, this may not be the case when using secondary data.

**Additional Revisions**

1. We revised the introduction using more recent sources.
2. We explained the method step by step.
3. We removed the Hosmer and Lemeshow tests from the method, as well as the corresponding findings, because in multilevel modeling, whether to evaluate whether the data is good or bad for the model, Tests of Heterogeneity is sufficient.
4. To have a better knowledge of multilevel modeling model comparison and adequate checking, we added the "**Model comparison, adequacy, and heterogeneity checking**" section in the methods part.
5. Since we previously displayed the odds ratio with the confidence interval for Z in the last table. Now, we therefore provided the odds ratio together with the corresponding confidence interval.
