# Supplementary material for: Prevalence and correlates associated with early childbearing among teenage girls in Ethiopia: A multilevel analysis
Source: PLoS One. 2023 Aug 8;18(8):e0289102. doi: 10.1371/journal.pone.0289102 (PMC10409268; doi:10.1371/journal.pone.0289102)
Supplement: Attachment — Submitted filename: Revised Manuscript clean copy.docx [file pone.0289102.s002.docx]

**Correlates and prevalence associated with early childbearing among teenage girls in Ethiopia: A Multilevel Analysis**

**Authors**: - Abebe Debu Liga**^1*^**, Adane Erango Boyamo**^2^**, Yasin Negash Jabir**^3^,** Akalu Banbeta Tereda**^3^**

**^1^**Department of Statistics, College of Natural and Computational Sciences, Wolkite University, Ethiopia **^2^**Department of Statistics, College of Natural and Computational Sciences, Wachemo University, Ethiopia

**^3^**Department of Statistics, College of Natural Science, Jimma University, Ethiopia

***Corresponding author (ADL)**

**E-mail**: [abe.debu@yahoo.com](mailto:abe.debu@yahoo.com)

**Abstract**

**Background**: Teenage childbearing remains a significant global health concern, and in nations with limited resources, it is the major cause of newborn and mother deaths. Early teenage childbearing is still Ethiopia's public health issue. Therefore, the goal of this study was to identify the correlates and prevalence of influencing early childbearing among teenage girls across Ethiopia.

**Methods**: We conducted a secondary analysis of cross-sectional data from the 2016 Ethiopian Demographic and Health Survey. To determine the significantly correlated factors that influence adolescent pregnancy, a multilevel binary logistic regression analysis was used. The factors that have a significant association with early childbearing were identified using the Adjusted Odds Ratio (AOR) and 95% Confidence Interval (CI).

**Results**: The odds of an early childbearing among teenage girls was increased with first marriages occurring before the age of 18, non-formal education, being from a lower- or middle-class family, not using contraceptives, following Muslim or other religious beliefs, and being aware of the fertile window. Teenagers who had exposure to the media, however, had a reduced chance of becoming pregnant early.

**Conclusions**: The study indicates that early teenage childbearing is still Ethiopia's most significant public health problem. Therefore, the Ethiopian government should ban early marriage while also taking steps to reduce the risk through formal education, improved access to reproductive health education, and contraception, particularly for adolescent girls from low-income families.

**Keywords**: Early childbearing, Correlates, teenage girls, Random slope multilevel model, Ethiopia

**Introduction**

Childbearing among girls between the ages of 10 and 19 is known as a teenage pregnancy [1]. The United Nations Children Fund (UNICEF) also describes adolescent pregnancy as “a teenage girl, typically between the ages of 13 and 19 becoming pregnant” [2]. Teenage childbearing remains a serious concern and health issue for worldwide, and it is the main cause of newborn and mother death in nations with poor infrastructure [3]. In low- and middle-income countries, pregnancy-related problems are the leading cause of death for girls between the ages of 15 and 19 [4]. These women have a double the risk of dying during pregnancy and childbirth compared to women in their twenties, while the risk is five times higher for those under the age of 15 [5]. Teenagers are often inexperienced about the risks of sexual activity and reproductive health [6].

Pregnancy during teenage years has a number of adverse effects, including preterm labor and low birth weight [3, 7], neonatal death, obstructed labor, genital fistula and eclampsia, as well as the emotional and physical challenges of pregnancy [8- 10]. The reproductive health of women is additionally affected by unsafe abortion, sexually transmitted infections like HIV/AIDS, sexual violence, abortion, and restricted access to medical care [11]. Pregnancy among teens seems to be a global problem. Despite the fact that rates of teenage pregnancy have declined in most high-income countries since the mid-1990s [12], but they remain a substantial public health concern, particularly in Africa [13].

Africa has a higher rate of teenage pregnancies than the rest of the world [14-15], accounts for 18.8% of all teenage pregnancies, with 19.3% of those occurring in the sub-Saharan region [15]. In seven nations, including Ethiopia, more than 50% of all teenage births take [16]. In low- and middle-income nations, estimated 21 million and 12 million girls between the ages of 15 and 19 become pregnant and give birth every year respectively [15]. Studies have shown that reducing the high rate of adolescent pregnancy in developing countries can lower the high level of morbidity and mortality among mothers and newborns [17].

Nevertheless, the Ethiopian government has made reducing the risk of teen pregnancy one of its main priorities for sustainable development [18]. However, Ethiopia has a comparable situation to other sub-Saharan countries in terms of the prevalence of teenage childbearing. Approximately 16% of teenage pregnancies occurred, according to the EDHS-2016 report, with those living in rural areas experiencing a greater pregnancy rate [19]. Additionally, Mamo et al. predict that the prevalence of teenage pregnancies in Ethiopia was 23.59% in 2021 [20]. A 2011 WHO study findings states that teenage girl who fall pregnant before the age of 18 are 60% more likely to lose their child during the first few years of life [21] and are substantially more likely to face domestic violence [22].Previous studies identified that early sexual activity [23-24], early marriage [25], marital status [26-27, 30], education attainment [28-31], religion [32, 33, 42], age [26, 27, 30, 32, 34], place of residence [30, 32, 34, 35], household wealth [23, 24, 29], employment [27, 30], media exposure [31, 32, 36, 37], alcohol consumption [38] rape [13], contraceptive use [23, 24, 30, 32, 34, 39], lack of comprehensive sexuality education [40], and influence or pressure from peers [32] are all significantly associated with correlates of teenage pregnancy.

Though there are relatively few studies which look at teen pregnancies in Ethiopia as an entire phenomenon. The majority of the previously conducted studies about adolescent pregnancies are small-scale or institutional-based or specific to some area, those lack national-level information as well as a clear breakdown of the factors that affect teenage pregnancies. To overcome these problems, and in order to aid policy makers, program administrators, and health care authorities in making better planning and problem-solving decisions, this study intended to identify the correlates of influencing early childbearing among teenage girls across the nationwide level on basis of Multilevel analysis.

**Methods**

**Data Source and sampling procedure**

The present study utilized the data of the 2016 Ethiopian Demographic and Health Survey, which had been carried out in Ethiopia between January 18 and June 27, 2016 by the Central Statistical Agency. The survey has been carried out all over Ethiopia utilizing the community-based multistage stratified cluster sampling approach with two stages, including 645 counting areas. The study's sampled locations were split into two groups: 28 households in each cluster in the second phase and 202 urban and 443 rural areas in the first phase. The interview covered all females between the ages of 15 and 49. The survey covered 16,583 women in total, 3,498 of whom provided their information for the present study [19].

**Study variables**

The status of early childbearing among teenage girls was the response variable used in this study, and it was coded as "1" if the teenager became pregnant at an early age and "0" when they did not. While, age at first marriage, residence, educational attainment, region, household wealth, knowledge of fertility window, mass media exposure, employment status, religion, contraceptive uses, and alcohol consumption were all independent variables taken into consideration for this study.

**Inclusion and exclusion criteria**

Teenage girls under the age of nineteen who had complete information regarding early childbearing at the period of collecting data were included, while those under the age of 19 who were lacking in this information weren't included in the study. **Statistical analysis**

Each variable are presented along with their absolute and relative frequencies. They were cross-tabulated according to whether teenage girls had children early (yes/no), and the relationship to each independent variable was examined using chi-square tests. In these univariate analyses, variables that were significant at the 5% level were included in a multilevel logistic regression model with the status of early childbearing among teenage girls as the outcome. 95% confidence intervals and odds ratios have been established for the explanatory variables. All analyses were conducted using SPSS 21.0 (IBM Corp., Armonk, NY) and STATA 23 version.

**Multilevel logistic regression model**

A multilevel logistic regression model is an extension of a single-level logistic regression model that includes random effects in studies with nested data structured by more than one level [41]. Individuals at a lower level that are nested within integrated units at a higher level frequently serve as the focus of the analysis. Teenagers were taken as level 1 in this study's two-level logistic regression model, whereas regions were considered level 2 in order to identify unexplained variance both within and between groups. In this study, three multilevel logistic regression models a null model (model without predictors) or no explanatory variable which assessed the overall variance in the factors affecting childbearing within regions were examined hierarchically. Only variables at the individual level were considered in model 2. The combined effects of variables at the individual and regional levels were assessed by Model-3.

**Model comparison, adequacy and heterogeneity checking**

The multilevel logistic regression model must first be determined to be appropriate for the data set. Therefore, we applied the standard chi-square test for the contingency to determine whether or not there is systematic variation between the groups or regions. The most common techniques for assessing the degree of accuracy of fit in multilevel logistic regression models were deviance, AIC, and BIC values after it had been demonstrated that the data were appropriate for the model. The null and random coefficient models' deviation, AIC, and BIC scores provide the best model for predicting the prevalence of early teenage childbearing. Additionally, the intra-class correlation coefficient (ICC) quantifies the percentage of variance in the outcome variable that can be accounted for by the grouping structure. The clustered nature of the data and variation within and across communities were taken into account by assuming that each community possessed a distinct intercept and fixed coefficient.

**Ethics approval**

Secondary data sets from the 2016 EDHS, which were previously collected and held in confidence, were utilized in this analysis. Therefore, since we did not directly gather data from individuals, recruitment and involvement of participants were not necessary. The datasets can be accessed with the permission of the Measure DHS program and the Ethiopian Public Health Institute.

**Results**

**Descriptive Statistics Results**

This study made up 3498 teenagers from nine regional states and two administrative cities in Ethiopia. Among them, 10.26% were early pregnant or childbearing during the period of gathering the data. The majority of teenage girls were found in Affar (20.68%), Gambela (15.57%), and Somali (15.36%), as opposed to Addis Ababa (1.85%). Also majorities (13.59%) were from rural areas, and 14.84% were followed Muslim faiths. Out of all the teenage girls, 22.46% had no formal education at all, 9.47% completed elementary school, 4.64% had attended secondary school, and 1.94% had attended higher education. Similarly, 14.13 percent, 11.46 percent, and 4.87 percent of teenagers had been married for the first time when they were aged 15 to 17, 15 to 17, and older than 18 respectively. The greatest proportion of early childbearing was experienced by teenagers who were less than 15 years at their first marriage (table 1).

According to the summary, 17.61% of teenagers come from low-income families, followed by 12.13% from middle-class families and 4.93% from wealthy households. A majority of teenagers (18.14%) were aware that they were most fertile at the end of their menstrual cycle, and 41.23% of them used modern contraceptives. Similarly, a majority of teenagers (18.14%) were aware that they were most fertile at the end of their periods, 41.23% of them used modern contraception, 10.71% were unemployed, and 14.88% were not exposed to the media (table 1).

**Table 1:** Socio-demographic characteristics of childbearing status among teenage girls

|  |  |  | **Experience of Early childbearing** | | | **Chi-square test** | | |
| --- | --- | --- | --- | --- | --- | --- | --- | --- |
| **Variables** | **Categories** | Total | No | Yes | Rate | Value | df | P-value |
| Region | Tigray | 423 | 388 | 35 | 8.27% | 101.88 | 10 | ≤0.001 |
|  | Afar | 266 | 211 | 55 | 20.68% |  |  |  |
|  | Amhara | 355 | 330 | 25 | 7.04% |  |  |  |
|  | Oromia | 415 | 358 | 57 | 13.73% |  |  |  |
|  | Somali | 319 | 270 | 49 | 15.36% |  |  |  |
|  | Benishangul | 237 | 212 | 25 | 10.55% |  |  |  |
|  | SNNPR | 391 | 364 | 27 | 6.91% |  |  |  |
|  | Gambela | 212 | 179 | 33 | 15.57% |  |  |  |
|  | Harari | 183 | 157 | 26 | 14.21% |  |  |  |
|  | Addis Ababa | 432 | 424 | 8 | 1.85% |  |  |  |
|  | Dire Dawa | 265 | 246 | 19 | 7.17% |  |  |  |
| Place of Residence | Urban | 1246 | 1193 | 53 | 4.25% | 75.89 | 1 | ≤0.001 |
|  | Rural | 2252 | 1946 | 306 | 13.59% |  |  |  |
| Age at first marriage | < 15 years | 665 | 571 | 94 | 14.13% | 96.42 | 2 | ≤0.001 |
|  | 15-17 years | 1210 | 1024 | 186 | 11.46% |  |  |  |
|  | ≥ 18 years | 1623 | 1544 | 79 | 4.87% |  |  |  |
| Religion | Orthodox | 1409 | 1335 | 74 | 5.25% | 71.499 | 2 | ≤0.001 |
|  | Muslim | 1435 | 1222 | 213 | 14.84% |  |  |  |
|  | Others | 654 | 582 | 72 | 11.01% |  |  |  |
| Education | No education | 570 | 442 | 128 | 22.46% | 128.57 | 3 | ≤0.001 |
|  | Primary | 2027 | 1835 | 192 | 9.47% |  |  |  |
|  | Secondary | 798 | 761 | 37 | 4.64% |  |  |  |
|  | Higher | 103 | 101 | 2 | 1.94% |  |  |  |
| Household wealth | Poor | 1204 | 992 | 212 | 17.61% | 128.54 | 2 | ≤0.001 |
|  | Middle | 470 | 413 | 57 | 12.13% |  |  |  |
|  | Rich | 1824 | 1734 | 90 | 4.93% |  |  |  |
| Knowledge of the fertile window | During period | 147 | 132 | 15 | 10.20% | 62.20 | 5 | ≤0.001 |
|  | After period | 656 | 537 | 119 | 18.14% |  |  |  |
|  | Midpoint of period | 648 | 592 | 56 | 8.64% |  |  |  |
|  | Before period | 264 | 237 | 27 | 10.23% |  |  |  |
|  | Every time | 785 | 707 | 78 | 9.94% |  |  |  |
|  | Don't know | 998 | 934 | 64 | 6.41% |  |  |  |
| Contraceptive uses | None | 3287 | 3015 | 272 | 8.28% | 233.84 | 1 | ≤0.001 |
|  | Modern method | 211 | 124 | 87 | 41.23% |  |  |  |
| Employment Status | No | 2623 | 2342 | 281 | 10.71% | 2.30 | 1 | 0.129 |
|  | Yes | 875 | 797 | 78 | 8.91% |  |  |  |
|  | Female | 1125 | 1016 | 109 | 9.69% |  |  |  |
| Mass Media Exposure | No | 1552 | 1321 | 231 | 14.88% | 64.684 | 1 | 0.008 |
|  | Yes | 1946 | 1818 | 128 | 6.58% |  |  |  |
| Alcohol consumption | No | 3418 | 3087 | 331 | 9.68% | 54.39 | 1 | ≤0.001 |
|  | Yes | 80 | 52 | 28 | 35% |  |  |  |

## Association with the status of childbearing among teenage girls were significant in univariate analysis for the following variables, age at first marriage, region, place of residence, educational attainment, household wealth, knowledge of the fertile window, contraceptive use, religion, alcohol consumption, and mass media exposure (Table 1).

## Multilevel logistic regression results

### Tests of Heterogeneity

The chi-square tests of heterogeneity value in Table 2 is 101.880 with a p-value less than 0.05, which is significant and indicates that there is heterogeneity between childbearing among teenage girls in region of Ethiopia. It is therefore appropriate to examine a data set using multilevel logistic regression.

**Table 2:** **Chi-Square** Tests of Heterogeneity

| Statistics | Value | Df | p-value |
| --- | --- | --- | --- |
| Pearson Chi-Square | 101.880 | 10 | p≤0.001 |

# Results of Multilevel Binary Logistic Regression Model

The deviance, Akaike information criterion (AIC), and Bayesian information criterion (BIC) values were used to select the best multilevel models among the three fitted models. A model with the lowest AIC and deviance scores would be the best one. Therefore, the random slope model (Model 3) has lower deviance and AIC values than both the empty model (Model 1) and the random intercept model (Model 2), suggesting that it is more accurate in predicting early childbearing girls in Ethiopia (Table 3). Educational attainment and use of contraceptives were the two factors that varied the most throughout the region compared to other variables and were suitable for analysis utilizing the multilevel random slope model. The null model from table 3 has an ICC of 0.1016, indicating that the grouping structure in higher-level units or regions can account for 10.16% of the variation in the prevalence of early childbearing, and 89.94% of the variation is explained within lower-level units.

**Table 3: Summary results of model selection criteria and ICC**

| **Class** | **Null model**  **(Model 1)** | **Random intercept model**  **(Model 2)** | **Random slope model**  **(Model 3)** |
| --- | --- | --- | --- |
| Deviance | 72.02(p≤0.001) | 409.7528(p≤0.001) | 30.0107(p≤0.001) |
| AIC | 2246.424 | 1864.671 | 1844.649 |
| BIC | 2258.744 | 1963.231 | 1974.008 |
| ICC | 0.1016461 |  |  |

Table 4 also revealed that there is significant heterogeneity or variance among the teen childbearing rates in the between regional states of Ethiopia, with the confidence interval of the estimate of the random intercept excluding zero. Furthermore, the confidence interval for the random slopes for contraceptive use and educational attainment excludes zero, demonstrating that the educational and contraceptive practices used by teenagers vary from region to region.

**Table 4**: Confidence interval of Random intercept and Random slope models

| Random effects Parameters  Region: Unstructured | Estimate | Std. Err. | [95% Conf. Interval] |
| --- | --- | --- | --- |
| Variance (contraceptive use) | 2.046 | 1.271 | [.606 6.910] |
| Variance(Educational attainment) | 0.194 | 0.134 | [.050 .751] |
| Variance ( cons) | 0.021 | 0.038 | [.001 .666] |

The random slope multilevel model reveals age at first marriage, education attainment, household’s wealth, contraceptives uses, knowledge of the fertile window, religion, and exposure to media as significantly (p<0.05) associated with the early childbearing among teenage girls (Table 5).

Early marriage has been significantly associated to higher rates of early childbearing. Responding individuals with a first marriage that was less than 15 years old had an odds ratio of 3.14 (95% CI: 2.39, 4.14) compared to the reference group with a first marriage that was greater than or equal to 18 years old, and participants with a first marriage that was between 15 and 17 years old had an odds ratio of 2.23 (95% CI: 1.59, 3.13).

Conversely, higher education significantly decreased the rate of early childbearing. The odds ratio for individuals with at least a primary education was 0.43 (95% CI: 0.28, 0.64) compared to the reference group of people with no formal education. Similarly, participants with at least a secondary education had odds ratios of 0.26 (95% CI: 0.12, 0.53), and participants with a higher education had odds ratios of 0.10 (95% CI: 0.02, 0.59), compared to the reference group with no formal education.

Early childbearing and participants' religious affiliation were significantly associated. Muslim religion participants had a higher likelihood of being pregnant earlier compared to Orthodox religion participants (OR: 2.83, 95% CI: 1.92, 4.17) and other religion participants had a higher chance to get pregnant earlier to Orthodox religion participants (OR: 2.27, 95% CI: 1.40, 3.69). Similarly, early pregnancy rates were significantly associated with household wealth.

Teen girls from lower household wealth backgrounds were more likely to have children early than those from higher wealth histories (OR: 2.88, 95% CI: 2.03, 4.09), and girls from middle household wealth backgrounds were more likely to have children early than those from higher wealth households (OR: 2.33, 95% CI: 1.55, 3.52). Participants who had access to mass media had a lower likelihood of practicing early childbearing than those who had no access to it (OR: 0.76, 95% CI: 0.56, 1.02). The use of contraceptives greatly reduced the rate of early childbirth.

The use of contraceptives significantly decreased the rate of early childbirth. The odds of early childbearing were higher among those who did not utilize contraceptive methods than among those who did (OR: 18.46, 95% CI: 6.89, 49.49). Furthermore, the results suggest that participants' knowledge of the fertile window greatly boosted their likelihood of becoming pregnant early (Table 5).

**Table 5:** Random slope multilevel logistic results of factors associated with teenage childbearing

| **Variable** | **Category** | **AOR** | **95% Conf. Interval** | **P-value** |
| --- | --- | --- | --- | --- |
| Age at first marriage | ≥18 years (Ref.) |  |  |  |
|  | 15-17 years | 2.23 | (1.59, 3.13) | 0.000 |
|  | < 15 years | 3.14 | (2.39, 4.14) | 0.000 |
| Education | No education (Ref.) |  |  |  |
|  | Primary |  | (0.28, 0.64) | 0.000 |
|  | Secondary | 0.26 | (0.12, 0.53) | 0.000 |
|  | Higher | 0.10 | (0.02, 0.59) | 0.026 |
| Religion | Orthodox (Ref.) |  |  |  |
|  | Muslim | 2.83 | (1.92, 4.17) | 0.000 |
|  | Other | 2.27 | (1.40, 3.69) | 0.000 |
| Household wealth | Rich (Ref.) |  |  |  |
|  | Middle | 2.33 | (1.55, 3.52) | 0.000 |
|  | Poor | 2.88 | (2.03, 4.09) | 0.000 |
| Media Exposure | No (Ref.) |  |  |  |
|  | Yes | 0.76 | (0.56, 1.02) | 0.049 |
| Contraceptives use | Yes (Ref.) |  |  |  |
|  | No | 18.46 | (6.89, 49.49) | 0.000 |
| Knowledge of fertile window | Don’t know (Ref.) |  |  |  |
|  | During period | 2.60 | (1.32, 5.15) | 0.006 |
|  | After period | 4.56 | (3.17, 6.56) | 0.000 |
|  | Midpoint of period | 2.90 | (1.89, 4.46) | 0.000 |
|  | Before period | 2.98 | (1.71, 5.20) | 0.000 |
|  | Every time | 2.07 | (1.42, 3.02) | 0.000 |
| Constant |  | 0.02 | (0.01, 0.04) | 0.000 |

**Discussion**

This study sought to find the correlates of influencing early childbearing among teenage girls across Ethiopian regions. In this study, 10.26% of participants had children at early age during the period of gathering the data. The prevalence of teenager childbirth in this study is lower than that of a study conducted by Kassa et al. [15], which found that the rate was 18.8% in Africa and 19.3% in sub-Saharan African nations. There are regional variations in the rate of early childbearing. Girls from the Afar, Gambella, Somali, and Harari areas had higher rates, while Addis Ababa had the lowest the rate across Ethiopia's regions. This is probably due to regional differences in cultural and religious perspectives on sexuality, marriage, and reproduction.

According to this study, girls who got married early had an increased chance of having children early. This finding is in line with study by Presler-Marshall and Jones [25], which indicated that early marriage increased the risk of teenager childbirth, as well as with earlier studies that showed that early sexual activity increased the likelihood of early childbearing [23–24]. This might be because early marriage leads to multiple births in less than 24 months, repeated unplanned pregnancies, pregnancy terminations, and premature sterility due to lack of access to contraception. As a result, having children at an early age puts both the mother's and the child's health in danger and increases the likelihood that an infant may pass away in the first year of life [21].

Higher education also significantly reduces the rate of early childbirth. This is in agreement with studies conducted by Habito et al [28], Baba and Fujiwara [29], Eyasu [30], Worku et al [31], and Nalenga [32] that found a higher prevalence of teenage births among teenagers with no formal education. Similar to that, CSA, 2016 revealed that having more education had been associated to a lower risk of being exposed to teen pregnancies [19].

Teenage girls from lower and middle-income families were more likely to get pregnancies early than those from higher-income families. The findings fall in line with the studies done by Baba [29], Morón-Duarte [23], and Santos [24], which found that teenage girls who lived with poor parents were more likely to become pregnant early. The possible reason might be teenagers from low-income societies may not have enough access to knowledge about sexual and reproductive health issues, which increases their risk of having children at an earlier age.

The study found that respondents from the Muslim religion were more likely to become pregnant earlier. This finding is in agreement with a study conducted by Hossain [33], that reported Muslim women give birth earlier and have more children overall than women with non-Muslim partners. The finding also corresponds with a study conducted by Nalenga [32] that found Muslims requires more children than Christians.

Teenagers who obtained information from media sources including newspapers, radio, and television were less likely to become pregnant early than those who were not exposed to the media. This finding coincides with that of other studies done by Fatema [36], and Luchuo [37], which found that teenagers who were exposed to mass media had a lower chance to become pregnant early. The most likely explanation is that teenage girls are more likely to effectively use maternal health care when they are exposed to a variety of mass media.

This study found teenage girls who did not use contraceptive methods had a greater probability of having children early. The findings correspond with a study conducted by Morón-Duarte et al [23], Santos [24], and Ayele et al [39], which found that teenager childbirth is increased by a lack of contraceptive information and use.

Furthermore, teenagers who were aware of the fertile window during the menstrual cycle had an increased chance of becoming pregnant early. This finding contrasts with a study by Mezmur [42], who found that teenagers who were unaware of the fertile period of the menstrual cycle had a higher prevalence of childbirth.

**Conclusion**

The study reveals that 10.26% of teenagers nationwide have children at an early age; it shows that early teenage childbearing is still Ethiopia's worst public health issue. Age at first marriage before 18 years, non-formal education, lower- and middle-class household backgrounds, not using contraceptive methods, followers of Muslim and other religions, no access to media, and knowledge of the fertile window were all correlates of early teenage childbearing in the study. Therefore, the government should really outlaw early marriage while implementing measures to lower the risk through formal education, enhanced access to reproductive health education, and contraception, especially for teenage girls from the lowest-income families.

**Strength and limitations of the study**

The study had several strengths. First, it was based on data that were nationally representative, and second, it may provide policymakers and program planners with information that would help them develop effective intervention strategies at both the national and regional levels. Thirdly, the EDHS data are hierarchical, so the multilevel analysis was employed to account for this and obtain accurate estimates and standard errors. The EDHS survey was based on respondents' self-reports, which may have giving rise to recall bias, which is one weakness of this study. Additionally, because this study was based on secondary data, it is missing several crucial factors that could affect the outcome variable.

**Abbreviations**

AIC: Akaike Information Criterion; BIC: Bayesian Information Criterion; CSA: Central Statistical Agency; EDHS: Ethiopian Demographic and Health Survey; HIV: Human Immune Virus; ICC: Intra-class Correlation Coefficient; OR: Odds Ratio; SSAR: Sub-Saharan African regions; UNICEF: United Nations Children Fund; and WHO: World Health Organization

**Acknowledgments**

We really appreciate that the Ethiopian Central Statistical Agency (CSA) provided the EDHS dataset. The authors also expressed grateful to every expert and research assistants who contributed to the study, as well.

**Authors' contributions**

Abebe Debu Liga, Adane Erango Boyamo, Yasin Negash Jabir, Akalu Banbeta Tereda all contributed to the study's conception, data curation, resource, software, design, coordination, data analysis, interpretation, and write-up. Abebe Debu Liga wrote the manuscript. All authors read the manuscript preceding consenting to its submission for peer review. Abebe Debu Liga Yasin Negash Jabir, Akalu Banbeta Tereda made revisions to the manuscript. All authors have read and approved the final draft.

### Financial Disclosure Statement

The authors received no specific funding for this work. **References**

1. Ganchimeg T, Ota E, Morisaki N, Laopaiboon M, Lumbiganon P, Zhang J, Yamdamsuren B, Temmerman M, Say L, Tunçalp Ö, Vogel JP. Pregnancy and childbirth outcomes among adolescent mothers: a World Health Organization multicountry study. BJOG: An International Journal of Obstetrics & Gynaecology. 2014 Mar; 121:40-8. PMID: 24641534
2. United Nations Children’s Fund, UNICEF. Report on the regional forum on adolescent pregnancy, child marriage and early union in south-east Asia and Mongolia [internet]. 2018. Available from: <https://www.unicef.org/eap/media/3696/file/Adolescent%20pregnancy.pdf>
3. Neal S, Matthews Z, Frost M, Fogstad H, Camacho AV, Laski L. Childbearing in adolescents aged 12–15 years in low resource countries: a neglected issue. New estimates from demographic and household surveys in 42 countries. Acta obstetricia et gynecologica Scandinavica. 2012 Sep; 91(9):1114-8. PMID: 22620274
4. Grønvik T, Fossgard Sandøy I. Complications associated with adolescent childbearing in Sub-Saharan Africa: A systematic literature review and meta-analysis. PloS one. 2018 Sep 26; 13(9):e0204327. PMCID: PMC6157872, PMID: [30256821](https://pubmed.ncbi.nlm.nih.gov/30256821)
5. Omar K, Hasim S, Muhammad NA, Jaffar A, Hashim SM, Siraj HH. Adolescent pregnancy outcomes and risk factors in Malaysia. International Journal of Gynecology & Obstetrics. 2010 Dec 1;111 (3):220-3. PMID: 20800837
6. Simegn A, Azale T, Addis A, Dile M, Ayalew Y, Minuye B. Youth friendly sexual and reproductive health service utilization among high and preparatory school students in Debre Tabor town, Northwest Ethiopia: A cross sectional study. PloS one. 2020 Sep 30; 15(9):e0240033. PMID: 33206723 PMCID: PMC7673529
7. Rasheed S, Abdelmonem A, Amin M. Adolescent pregnancy in upper Egypt. International Journal of Gynecology & Obstetrics. 2011 Jan 1; 112(1):21-4. PMID: 21055750
8. Abbas AM, Ali SS, Ali MK, Fouly H, Altraigey A. The maternal and neonatal outcomes of teenage pregnancy in a tertiary university hospital in Egypt. Proceedings in Obstetrics and Gynecology. 2017 Oct 1; 7(3).
9. Andargie AA, Debu A. Determinants of obstetric fistula in Ethiopia. African health sciences. 2017 Sep 18;17(3):671-80. PMID: 29085394, PMCID: PMC5656212
10. Ayuba II, Gani O. Outcome of teenage pregnancy in the Niger Delta of Nigeria. Ethiopian journal of health sciences. 2012; 22(1):45-50. PMID: 22984331 PMCID: PMC3437979
11. Joesephine P, Premraj C. Adolescent sexual and reproductive health. Global Journal for Research Analysis. 2016; 5(5).
12. Sedgh G, Finer LB, Bankole A, Eilers MA, Singh S. Adolescent pregnancy, birth, and abortion rates across countries: levels and recent trends. Journal of adolescent health. 2015 Feb 1; 56(2):223-30. PMID: 25620306,  PMCID: PMC4852976
13. Yakubu I, Salisu WJ. Determinants of adolescent pregnancy in sub-Saharan Africa: a systematic review. Reproductive health. 2018 Dec; 15(1):1-1. PMID: 29374479, PMCID: PMC5787272
14. Darroch JE, Woog V, Bankole A, Ashford LS. Adding it up: costs and benefits of meeting the contraceptive needs of adolescents.
15. Kassa GM, Arowojolu AO, Odukogbe AA, Yalew AW. Prevalence and determinants of adolescent pregnancy in Africa: a systematic review and meta-analysis. Reproductive health. 2018 Dec; 15(1):1-7. PMID: 30497509, PMCID: PMC6267053
16. Braine T. Adolescent pregnancy: a culturally complex issue. World Health Organization. Bulletin of the World Health Organization. 2009 Jun 1; 87(6):410. PMID: 19565115, PMCID: PMC2686202
17. Nove A, Matthews Z, Neal S, Camacho AV. Maternal mortality in adolescents compared with women of other ages: evidence from 144 countries. The Lancet Global Health. 2014 Mar 1; 2 (3):e155-64. PMID: 25102848
18. Ayele BG, Gebregzabher TG, Hailu TT, Assefa BA. Determinants of teenage pregnancy in Degua Tembien District, Tigray, Northern Ethiopia: A community-based case-control study. PloS one. 2018 Jul 25; 13(7):e0200898. PMID: 30044850, PMCID: PMC6059451
19. Central Statistical Agency (CSA) Ethiopia and ICF. *Ethiopia Demographic and Health Survey 2016*. Addis Ababa, Ethiopia, and Rockville, Maryland, USA: CSA and ICF; 2016. Available from: <https://dhsprogram.com/pubs/pdf/FR328/FR328.pdf>
20. Mamo K, Siyoum M, Birhanu A. Teenage pregnancy and associated factors in Ethiopia: a systematic review and meta-analysis. International Journal of Adolescence and Youth. 2021 Jan 1; 26(1):501-12.
21. World Health Organization. Maternal, newborn, child and adolescent health. 2016.
22. Loaiza E, Liang M. Adolescent pregnancy: a review of the evidence. UNFPA; 2013.
23. Morón-Duarte LS, Latorre C, Tovar JR. Risk factors for adolescent pregnancy in Bogotá, Colombia, 2010: a case-control study. Revista Panamericana de Salud Pública. 2014 Sep; 36(3):179-84. PMID: 25418768
24. Santos MI, Rosario F. A score for assessing the risk of first-time adolescent pregnancy. Family practice. 2011 Oct 1; 28(5):482-8. PMID: 21525140
25. Presler-Marshall E, Jones N. Empowering girls to prevent early pregnancy. Oversees Development Institute. 2012 Jul.
26. Ayele BG, Gebregzabher TG, Hailu TT, Assefa BA. Determinants of teenage pregnancy in Degua Tembien District, Tigray, Northern Ethiopia: A community-based case-control study. PloS one. 2018 Jul 25; 13(7):e0200898. PMID: 30044850, PMCID: PMC6059451
27. Nwosu UM. Contemporary factors of teenage pregnancy in rural communities of Abia state, Nigeria. International Journal of Community Medicine And Public Health. 2017 Feb; 4(2):588-92.
28. Habito CM, Vaughan C, Morgan A. Adolescent sexual initiation and pregnancy: what more can be learned through further analysis of the demographic and health surveys in the Philippines? BMC public health. 2019 Dec; 19(1):1-3. PMID: 31429733, PMCID: PMC6701073
29. Baba S, Iso H, Fujiwara T. Area-level and individual-level factors for teenage motherhood: a multilevel analysis in Japan. PloS one. 2016 Nov 10; 11(11):e0166345. PMID: 27832177, PMCID: PMC5104437
30. Eyasu AM. Determinants of adolescent fertility among Rural Women of Ethiopia. Open Access Library Journal. 2016 Mar 31;3(3):1-9.
31. Worku MG, Tessema ZT, Teshale AB, Tesema GA, Yeshaw Y. Prevalence and associated factors of adolescent pregnancy (15–19 years) in East Africa: a multilevel analysis. BMC Pregnancy and Childbirth. 2021 Dec; 21:1-8. PMID: 33771106, PMCID: PMC7995759
32. Nalenga GZ. *Causes of unintended pregnancy among adolescents in Addis Abeba, Ethiopia* (Master's thesis, Norwegian University of Life Sciences, Ås).
33. Hossain M. Women’s education, religion and fertility in Bangladesh. 2017
34. Ayanaw Habitu Y, Yalew A, Azale Bisetegn T. Prevalence and factors associated with teenage pregnancy, Northeast Ethiopia, 2017: a cross-sectional study. Journal of pregnancy. 2018 Nov 1; 2018. PMID: 30515326, PMCID: PMC6236922
35. Sayem AM, Nury AT. Factors associated with teenage marital pregnancy among Bangladeshi women. Reproductive health. 2011 Dec; 8(1):1-6. PMID: 21599904, PMCID: PMC3187734
36. Fatema K, Lariscy JT. Mass media exposure and maternal healthcare utilization in South Asia. SSM-Population Health. 2020 Aug 1; 11:100614. PMID: 32596437, PMCID: PMC7306581
37. Bain LE, Muftugil-Yalcin S, Amoakoh-Coleman M, Zweekhorst M, Becquet R, de Cock Buning T. Decision-making preferences and risk factors regarding early adolescent pregnancy in Ghana: stakeholders’ and adolescents’ perspectives from a vignette-based qualitative study. Reproductive health. 2020 Dec; 17(1):1-2. PMID: 32917278, PMCID: PMC7488420
38. Panova OV, Kulikov AM, Berchtold A, Suris JC. Factors associated with unwanted pregnancy among adolescents in Russia. Journal of pediatric and adolescent gynecology. 2016 Oct 1; 29(5):501-5. PMID: 27108227
39. Ayele BG, Gebregzabher TG, Hailu TT, Assefa BA. Determinants of teenage pregnancy in Degua Tembien District, Tigray, Northern Ethiopia: A community-based case-control study. PloS one. 2018 Jul 25; 13(7):e0200898. PMID: 30044850, PMCID: PMC6059451
40. Atuyambe LM, Kibira SP, Bukenya J, Muhumuza C, Apolot RR, Mulogo E. Understanding sexual and reproductive health needs of adolescents: evidence from a formative evaluation in Wakiso district, Uganda. Reproductive health. 2015 Dec; 12(1):1-0. PMID: 25896066, PMCID: PMC4416389
41. Bosker R, Snijders TA. Multilevel analysis: An introduction to basic and advanced multilevel modeling. Multilevel analysis. 2011:1-368.
42. Mezmur H, Assefa N, Alemayehu T. Teenage pregnancy and its associated factors in eastern Ethiopia: a community-based study. International Journal of Women's Health. 2021 Feb 26:267-78. PMID: 33664597, PMCID: PMC7924244
