# Supplementary material for: Prevalence and correlates associated with early childbearing among teenage girls in Ethiopia: A multilevel analysis
Source: PLoS One. 2023 Aug 8;18(8):e0289102. doi: 10.1371/journal.pone.0289102 (PMC10409268; doi:10.1371/journal.pone.0289102)
Supplement: Attachment — Submitted filename: R2-Rebuttal Letter.docx [file pone.0289102.s003.docx]

PONE-D-22-18542R1

Correlates and prevalence associated with early childbearing among teenage girls in Ethiopia: A Multilevel Analysis

PLOS ONE

**Title Modification**: We interchanged the word “Correlates and prevalence” by “Prevalence and correlates” in the title.

Additional Editor Comments:

- There is no clear description why you are intended to this study.

**Author’s response**: We elaborated more on it per the editorial comment in the last paragraph of the introduction.

- What are the gaps you have seen from the previous studies?

**Author’s response**: We elaborated more on it per the editorial comment in the last paragraph of the introduction.

- What does it mean teenage pregnancy?

**Author’s response**: We defined it on the first two citations of the introduction of this study.

- If it is pregnancy before the age of 18 years, how did you talk about marriage before 18 considered as a significant factor?

**Author’s response**: Dear editor, our study is only based on teenage girls under the age of nineteen, as we have pointed out in the exclusion and exclusion criteria. Even though marriage under 18 is illegal in Ethiopia, Ethiopian demographic and Health survey data show that most Ethiopian girls marry under the age of 18.  As a result, marriage before 18 can be a significant factor associated with teenage childbearing.

-Can we assess prevalence by Odds ratio?

**Author’s response**: No, we can assess only the correlates (the associated factors) of teenage childbearing by Odds ratio. We only used the prevalence in the title to show the prevalence rate of early teenage childbearing, and we added the prevalence in the result of the abstract.

-You have assessed the associated factors, so how can you know about the burden/ prevalence of teenage pregnancy?

**Author’s response**: We also included its prevalence, as stated in the study's title. We incorporated the prevalence rate in the abstract and reported it as 10.26%.

- There is no clear description about the sample size, procedure of extraction the participants.

**Author’s response**: Amended per the editor comments.

- The other challenge in your result is you identified non modifiable factors like religion as significant factor, what will be your recommendation?

**Author’s response**: We have tried to elaborate on the possible explanations in the discussion, and conclusion. 
- The recommendation is not based on your results.
**Author’s response**: We have tried to modify it.

Reviewers' comments:
Reviewer's Responses to Questions

**Comments to the Author**
1. If the authors have adequately addressed your comments raised in a previous round of review and you feel that this manuscript is now acceptable for publication, you may indicate that here to bypass the “Comments to the Author” section, enter your conflict of interest statement in the “Confidential to Editor” section, and submit your "Accept" recommendation.

Reviewer #2: All comments have been addressed

Reviewer #3: (No Response)

2. Is the manuscript technically sound, and do the data support the conclusions?

The manuscript must describe a technically sound piece of scientific research with data that supports the conclusions. Experiments must have been conducted rigorously, with appropriate controls, replication, and sample sizes. The conclusions must be drawn appropriately based on the data presented.

Reviewer #2: Yes

Reviewer #3: Partly

3. Has the statistical analysis been performed appropriately and rigorously?

Reviewer #2: Yes

Reviewer #3: Yes

4. Have the authors made all data underlying the findings in their manuscript fully available?

The [PLOS Data policy](http://www.plosone.org/static/policies.action#sharing) requires authors to make all data underlying the findings described in their manuscript fully available without restriction, with rare exception (please refer to the Data Availability Statement in the manuscript PDF file). The data should be provided as part of the manuscript or its supporting information, or deposited to a public repository. For example, in addition to summary statistics, the data points behind means, medians and variance measures should be available. If there are restrictions on publicly sharing data—e.g. participant privacy or use of data from a third party—those must be specified.

Reviewer #2: Yes

Reviewer #3: Yes

5. Is the manuscript presented in an intelligible fashion and written in standard English?

PLOS ONE does not copyedit accepted manuscripts, so the language in submitted articles must be clear, correct, and unambiguous. Any typographical or grammatical errors should be corrected at revision, so please note any specific errors here.

Reviewer #2: Yes

Reviewer #3: No

6. Review Comments to the Author

Please use the space provided to explain your answers to the questions above. You may also include additional comments for the author, including concerns about dual publication, research ethics, or publication ethics. (Please upload your review as an attachment if it exceeds 20,000 characters)

Reviewer #2: I commend the authors for revising the manuscript and effecting changes at the various points appropriately.

Reviewer #3: Thanks authors as they provided response to previous comments, however, there are some comments that should be addressed by authors as follows:
Abstract:
Methods: Please write about instrument that you used for data collection. For instance how authors assessed media used by participants.

**Author’s response**: We used data from the Ethiopian Demographic and Health Survey, gathered by the Ethiopian Central Statistical Agency. We considered mass media exposure as watching television, listening to the radio, and reading a newspaper or magazine at least once a week. We incorporated it in the study variable section.

Results: please provide the prevalence of early childbearing teenage as you stated in the title of manuscript.

**Author’s response**: We have provided it per the reviewer’s comment.

Introduction

1. Readers like to know is child marriage legal in Ethiopia? What is the legal age for marriage?

According to Ethiopia's amended family code, getting married before reaching 18 is no longer acceptable.

**Author’s response**: In the introduction's fifth paragraph, we elaborated on it.

2. Please provide some information about health care of adolescents who got married for instance if they receive contraception and other cares.

**Author’s response**: In the end of introduction's fourth paragraph, we tried to elaborate about it.

Methods

1. As authors used data collection in 2016, readers would like to know the present situation of Ethiopia.

**Author’s response**: Due to a deficient national documentation system and the fact that past studies were conducted on a local scale, institutionally or regionally focused, there is no evidence available on the prevalence of early teen pregnancy in Ethiopia nowadays.

Results

1) Please provide the number of teenagers who were pregnant along with the percentage.

**Author’s response**: We have provided it in the second sentence of descriptive result as well as row 2 of table 1.

2) Please report the number and percentages of teenagers who were pregnant in age of <15 and between 15 and 19.

**Author’s response**: We have provided it in the third sentence of descriptive result.

3) Page 16, there are two lines that are repetition of their earlier lines, please remove them.

**Author’s response**: Removed the repetition and reshaped it.

4) As in Table 1, authors stated the age in first marriage, I would like to know, did you have second marriage in your study? How many were pregnant without marriage?

**Author’s response**: The number of participants who were pregnant without marriage was not considered in our study.

5) Table 1: did you ask about non-modern contraception?

**Author’s response**: We did not gather the data ourselves. We used data from the Ethiopian Demographic and Health Survey, gathered by the Ethiopian Central Statistical Agency. They collected data on 16,583 women in total, but only 3,498 of those women provided all the necessary information. Also, they gathered data on non-modern methods of contraception, and the EDHS report showed that some respondents used them; however, when the missing data was cleared, none of the women who used non-modern methods of contraception were included.

6) The age difference between teenagers and their husbands may play a role in teenagers pregnancy, please report it along with their occupation.

**Author’s response**: We accepted this insightful recommendation.

However, we chose only eleven variables for consideration at the beginning of this study out of a huge number of variables in the Ethiopian Demographic and Health Survey data. Therefore, it is now challenging to include the husbands' age and work status at this point because doing so results in the responses of the women not being matched unless all variables are selected at the same time. In order to perform it now, it takes time. We hope that you can understand this problem.

7) All tables: please report p=0.000 as p<0.0001.

**Author’s response**: We amended it throughout the table per the reviewer comment.

**Discussion**

1. In Comparison the rate of teenage pregnancy in the present study with other studies, indicate the year of the study. This is good to know if the teenage pregnancy is in decrease.

**Author’s response**: We provided it in the document.

2. Please expand the results of the study regarding why Muslim teenagers have early pregnancy and more children?

**Author’s response**: We have tried to elaborate on the possible explanations in the discussion.
